# Development and characterization of SSR markers in *Phoebe zhennan*

**DOI:** 10.7717/peerj.20434

**Published:** 2025-12-15

**Authors:** Qing Liang, Yuhuan Jia, Ruxia Shi, Dan Zhao, Mei Luo

**Affiliations:** 1 College of Life Sciences/Institute of Agro-bioengineering, Guizhou University, Guiyang, Guizhou, China; 2 School of Biology and Engineering, Guizhou Medical University, Guiyang, Guizhou, China

**Keywords:** *Phoebe zhennan*, Genomic SSR, Molecular marker development, GD

## Abstract

*Phoebe zhennan* S. K. Lee & F. N. Wei is a valuable endemic species in China, widely recognized for its high-quality timber and ornamental value. It has been classified as an endangered species under national protection, with its primary distribution areas including Guizhou, Sichuan, and Hunan. Investigating the genetic diversity (GD) of this species plays a crucial role in developing effective strategies for its conservation and sustainable utilization. Nevertheless, the progress in molecular marker development has been constrained by the scarcity of reported reference genomes for *P. zhennan*. In this study, the genomic gap of the endangered and precious tree species *P. zhennan* was filled. The simple sequence repeat (SSR) markers of the *P. zhennan* genome were analyzed using magnetic bead enrichment technology. A total of 794,128 SSR loci were identified. From a random selection of 108 primer pairs, 20 primer pairs with high polymorphism and excellent stability were screened and subsequently utilized to assess the GD of 24 *Phoebe* populations comprising 174 individuals primarily from Guizhou Province. The results demonstrated that the SSR loci were predominantly dinucleotide repeats (accounting for 54.31%), among which the AG/CT motif exhibited the highest frequency. The polymorphism information content (PIC) of the 20 SSR primer pairs ranged from 0.777 to 0.903, with an average value of 0.859, reflecting their substantial polymorphism. The Shannon information index of the Guizhou *P. zhennan* population ranged from 1.196 to 1.928, with an average value of 1.518. The Nei’s genetic diversity index (H) varied between 0.661 and 0.832, averaging 0.743. The SN population exhibited relatively high genetic diversity, whereas the MT population demonstrated relatively low genetic diversity. Further investigation revealed that the populations in Gulin, Sichuan; Zigong, Sichuan; Tongnan, Chongqing; and Baijia, Hunan possessed high and stable genetic diversity values. Genetic differentiation analysis indicated that the genetic variation within the Guizhou *P. zhennan* population primarily stemmed from intra-population variation. Additionally, frequent gene flow was observed among populations, with alleles being widely distributed across populations. Based on the phylogenetic tree, the species *Phoebe bournei*, *Phoebe sheareri*, and *Phoebe chekiangensis*, as well as the populations of *P. zhennan* from different regions, were clearly differentiated. The SSR molecular markers developed in this study confirmed at the molecular level that these species represent distinct evolutionary lineages. These findings provide valuable SSR molecular markers and a robust scientific foundation for breeding programs, species identification, and genetic diversity studies of germplasm resources within the genus *Phoebe*.

## Introduction

*Phoebe zhennan S. K. Lee & F. N. Wei*, an endemic tree species within the *Phoebe* genus, is a critically endangered component of China’s unique flora. Classified as a second-grade nationally protected species in China and listed on the IUCN Red List of Threatened Species since 1984, *P. zhennan* faces significant conservation challenges. Its distribution is primarily restricted to fragmented populations in Sichuan, Guizhou, and Hunan provinces, with notable concentrations in Zunyi, Tongren, and Qiandongnan of Guizhou (Lu et al., 2018). This species is highly valued for its straight trunk, distinctive fragrance, and durable, hard material, making it significant in architecture, furniture, and handicrafts (Yang et al., 2022; Xu & Zang, 2023). The long-term survival of *P. zhennan* is threatened by multiple factors. Its slow growth rate, coupled with severe habitat degradation and historical over-exploitation, has led to a drastic decline in wild populations (Xiao et al., 2020). While frugivorous birds act as its primary seed dispersers, demographic studies suggest limitations in recruitment and dispersal efficiency likely exacerbate population vulnerability and genetic risks. This decline complicates species identification within the genus, as distinguishing morphological characters, particularly subtle leaf differences, become less reliable with dwindling and fragmented populations. Protecting such endemic, ecologically significant, and economically valuable *Lauraceae* species aligns with critical national and global conservation priorities.

Molecular markers offer powerful tools for conservation genetics, providing direct insights into species’ inherent genetic makeup unaffected by environmental variation. Among available markers, simple sequence repeats (SSRs) are particularly advantageous due to their high polymorphism, codominant inheritance, reproducibility, and cost-effectiveness, making them ideal for assessing genetic diversity (GD), constructing germplasm databases, resolving species identities and studying population genetic structures (Schlötterer, 2004; Lu et al., 2015; Su et al., 2023). Previous genetic studies on *P. zhennan* have primarily utilized EST (expressed sequence tag-simple sequence repeat, EST-SSR) based on transcriptome data. For example, (Shi et al., 2016) developed SSR markers from the transcriptome of Sichuan *P. zhennan*, identifying nine polymorphic loci. 14 SSR primer pairs were used to assess GD in germplasm resources and construct identity profiles. However, reliance on transcriptome-derived SSRs presents limitations: they are inherently biased towards expressed genomic regions, potentially overlooking substantial variation in non-coding DNA and providing incomplete genomic coverage. The lack of a publicly available reference genome for *P. zhennan* further constrains comprehensive marker development and broader applications, such as resolving persistent taxonomic uncertainties within the genus, such as *P. zhennan* and *Phoebe bournei* (*Hemsl.*) *Yang* remains controversial (Li et al., 2018; Ding et al., 2018).

Therefore, in this study, we aim to develop molecular markers and investigate the GD within the *Phoebe* genus. This is essential for enhancing understanding of genetic variation, fundamental to germplasm cultivation, species identification, and conservation efforts.

## Materials & Methods

### Plant material

The unique biogeographic transitional zone status and karst ecological environment of Guizhou Province have made it an important distribution area for endemic tree species such as *P. zhennan*. However, it also faces ecological vulnerability and conservation challenges, including severe habitat fragmentation. Based on the endangered conservation status of this species, its slow growth, ecological vulnerability caused by human pressure, and the biogeographic significance of Guizhou Province, research on *P. zhennan* has been conducted. The distribution range of wild *P. zhennan* in Guizhou was initially determined through consultation with the Chinese Virtual Herbarium (CVH, https://www.cvh.ac.cn/) and regional Chinese local flora. *P. zhennan* populations are primarily located near village settlements rather than in nature reserves.

Under the guidance and approval of the local Forestry Bureau, representative habitats of *P. zhennan* were selected for field investigations. At each sampling site, fresh and healthy leaves were collected from five to seven trees that were at least 50 m apart. The leaves were quickly frozen in liquid nitrogen and the geographical coordinates of the collection sites were recorded. The samples were then stored at −80 °C.

To assess the efficacy of the developed SSR (Simple Sequence Repeat) markers and conduct genetic diversity analysis, a total of 174 samples were collected from 24 populations across five provinces. Among these, the *P. zhennan* samples were predominantly collected from the Zunyi and Tongren regions of Guizhou Province. The specific collection sites encompassed: Daozhen (DZ), Tongzi (TZ), Jianhe (JH), Taijiang (TJ), Yuqing (YQ), Fenggang (FG), Chishui (CS), Dejiang (DJ), Jiangkou (JK), Sinan (SN), Meitan (MT), Shiqian (SQ), Wuchuan (WC), Xishui (XS) and Zhen’an (ZA). Additionally, samples from Zigong City (ZG), Luzhou City (LZ), and Gulin County (GL) in Sichuan Province, Baijia Town in Hunan Province (BJ), and Tongnan District in Chongqing Municipality (TN) were included for kinship analysis. To support taxonomic classification and species identification, this study also included samples of *Lindera megaphylla* Hemsl. (collected from Yanhe County, Guizhou Province), *P. bournei* (collected from Guizhou University) and *Phoebe chekiangensis* C.B. Shang (collected from Qingyuan County, Zhejiang Province). This study completed the classification and identification of *P. zhennan*. For detailed sample information, please refer to Table 1. The *P. zhennan* samples used for SSR-enriched library construction were collected from the Changlin Seed Orchard in Sichuan Province and were provided by Guizhou Academy of Forestry Sciences.

**Table 1:** Geographic coordinates of the sampling site.

Sampling site		Longitude (X)/Latitude (Y)	Accession	Amount
Dejiang County, Guizhou Province	DJ	107°52′54″E/28°13′37″N	DJ20-01, DJ20-02, DJ20-03, DJ20-04, DJ20-05, DJ20-06	6
Sinan County, Guizhou Province	SN	107°55′57″E/27°50′13″N	SN20-02, SN20-03, SN20-08, SN20-16, SN20-11, SN20-17, SN20-18, SN20-09, SN20-06	9
Shiqian County, Guizhou Province	SQ	108°08′21″E/27°26′52″N	SQ20-01, SQ20-03, SQ20-06, SQ20-05, SQ20-07, SQ20-13, SO20-17, SO20-21, SQ20-15, SQ20-22, SQ20-23	11
Jiangkou County, Guizhou Province	JK	108°46′58″E/27°38′14″N	JK20-01, JK20-02, JK20-03, JK20-04, JK20-05, JK20-06, JK20-07, JK20-08	8
Wuchuan County, GuizhouProvince	WC	107°51′35″E/28°22′13″N	WC20-01, WC20-02, WC20-03, WC20-04, WC20-05, WC20-06	6
Xishui County, Guizhou Province	XS	106°35′21″E/28°22′12″N	XS20-01, XS20-02, XS20-03, XS20-04	4
Zhengan County, Guizhou Province	ZA	107°25′57″E/28°32′14″N	ZA20-01, ZA20-02, ZA20-03, ZA20-04, ZA20-05, ZA20-06	6
Meitan County, Guizhou Province	MT	107°26′18″E/27°26′42″N	MT20-01, MT20-02, MT20-03, MT20-04	4
Chishui City, Guizhou Province	CS	106°28′57″E/28°25′22″N	CS20-01, CS20-02, CS20-03, CS20-04, CS20-05, CS20-06, CS20-07	7
Yuqing County, Guizhou Province	YQ	107°58′26″E/27°37′22″N	YQ22-01, YQ22-02, YQ22-03, YQ22-04, YQ22-05, YQ22-06, YQ22-07, YQ22-08, YQ22-09, YQ22-10, YQ22-11	11
Fenggang County, Guizhou Province	FG	107°51′51″E/27°56′59″N	FG22-01, FG22-02, FG22-03, FG22-04, FG22-05, FG22-06, FG22-07, FG22-08	8
Tongzi County, Guizhou Province	TZ	106°38′55″E/28°04′49″N	TZ23-01-1, TZ23-01-2TZ23-01-3, TZ23-02, TZ23-03, TZ23-04	6
Daozhen County, Guizhou Province	DZ	107°34′51″E/29°04′48″N	DZ23-01, DZ23-02, DZ23-03, DZ23-04, DZ23-05	5
Jianhe County, Guizhou Province	JH	108°37′15″E/26°32′29″N	JH23-01, JH23-02, JH23-03	3
Taijiang County, Guizhou Province	TJ	108°20′31″E/26°37′37″N	TJ23-01, TJ23-02, TJ23-03, TJ23-04, TJ23-05	5
Zhongjie Town, Guizhou Province	ZJ	108°32′26″E/28°29′05″N	DJ20-01, DJ20-02, DJ20-03, DJ20-04, DJ20-05, DJ20-06	6
Guiyang Botanical Garden, Guizhou Province	ZIN	106°40′12″E/26°33′36″N	ZIN23-01, ZIN23-02, ZIN23-03, ZIN23-04, ZIN23-05, ZIN23-06, ZIN23-07, ZIN23-08, ZIN23-09, ZIN23-10	10
Guizhou University, Guizhou Province	MN	106°40′05″E/26°23′06″N	MN23-01, MN23-02, MN23-03, MN23-04, MN23-05, MN23-06, MN23-07, MN23-08, MN23-09, MN23-10	10
**Total number of Guizhou Province**				**125**
Qingyuan County, Zhejiang Province	ZJN	119°03′36″E/27°37′12″N	ZJN23-01, ZJN23-02, ZJN23-03, ZJN23-04, ZJN23-05, ZJN23-06, ZJN23-07, ZJN23-08, ZJN23-09, ZJN23-10	10
**Total number of** ** Zhejiang Province**				**10**
Zigong City, Sichuan Province	ZG	104°24′35″E / 29°25′51″N	ZG23-01, ZG23-02, ZG23-03, ZG23-04, ZG23-05	5
Luzhou City, Sichuan Province	LZ	105°39′17″E/28°39′28″N	NM23-01, NM23-02, NM23-03, NM23-04, NM23-05, NM23-06, NM23-07, NM23-08	8
Gulin County, Sichuan Province	GL	105°43′25″E/28°11′17″N	GL23-01, GL23-02, GL23-03, GL23-04, GL23-05, GL23-06, GL23-07, GL23-08	8
**Total number of** ** Sichuan Province**				**21**
Baijia Town, Hunan Province	BJ	113°14′04″E/28°02′56″N	BJ23-01, BJ23-02, BJ23-03, BJ23-04, BJ23-05, BJ23-06, BJ23-07	7
**Total number of** ** Hunan Province**				**7**
Tongnan District, Chongqing	TN	105°44′12″E/29°50′31″N	TN23-01, TN23-02, TN23-03, TN23-04, TN23-05, TN23-06, TN23-07, TN23-08, TN23-09, TN23-10, TN23-11	11
**Total number of** ** Chongqing**				**11**
**Total**				**174**

### Methods

#### Extraction of DNA

Genomic DNA was extracted by modified cetyltrimethylammonium bromide (CTAB) technology (Tamari, Hinkley & Ramprashad, 2013; Zhao et al., 2021). Based on the traditional CTAB extraction buffer, some special antioxidants (*e.g.*, *β*-mercaptoethanol) were incorporated to prevent the oxidation of phenolic substances, and an appropriate amount of polyvinylpyrrolidone (PVP) was added to bind impurities such as polysaccharides. These modifications enhanced the purity of nucleic acids, thereby ensuring the integrity of the extracted DNA, uniform fragment size, and higher stability and reliability of the experimental results. The integrity of the DNA was detected by 0.8% agarose gel electrophoresis, while DNA concentration and purity were determined using a NanoDrop 2000 spectrophotometer (with the A_260_/A_280_ ratio controlled within the range of 1.8–2.0). All DNA samples were stored at −80 °C for future use.

#### Construction and sequencing of SSR-enriched libraries

DNA samples were sent to Shanghai Parson Biotechnology Co., Ltd. for SSR-enriched library construction and sequencing. The libraries were constructed based on the standard protocol of the Illumina TruSeq DNA Library Preparation Kit. Genomic DNA was randomly fragmented using a sonicator, followed by end-repair with End Repair Mix2. An “A” was added to the 3′ end of the fragments, and specific adapters were ligated. Small fragments (<300 bp) were removed using AMPure XP beads, and the target fragments of 300–500 bp were size-selected and purified. These fragments were then ligated to the adapter for PCR amplification. The prepared libraries were sequenced on the Illumina NovaSeq 6000 platform for paired-end 150 bp sequencing. FASTQ software was used to perform quality control on the raw sequencing data and remove low-quality data. Quality control procedures included: removing adapter sequences; trimming bases at the 5′ end with a sequencing quality value <20 or identified as N; trimming bases at the 3′ end with a sequencing quality value <3 or identified as N; removing reads with an N content >10%; discarding reads <30 bp in length. The number of clean reads, total base pairs, GC content, and Q30 ratio were calculated, and high-quality clean reads with Q30 ≥ 80% were retained. A total of 794,128 SSR loci were recognized in 1,642,465 sequences of the *P. zhennan* SSR-enriched library using MISA software (Table 2). The raw data have been submitted to the National Center for Biotechnology Information (NCBI), with the project number PRJNA1224921.

**Table 2:** SSR site search results statistics.

Sample	Numbers
The total number of sequences examined	1,642,465
The total size of examined sequences (bp)	551,443,009
The total number of identified SSRs	794,128
Number of SSR containing sequences	578,177
Number of sequences containing more than 1 SSR	162,869
The number of SSRs present in compound formation	178,046

#### Characterization of SSR loci and primer design

Based on the high-quality sequencing data, 10% of the samples were randomly selected for technical replicate sequencing to confirm that the genotyping error rate was below 1%. Sequences were assembled using the SOAPDenovo2 software, and SSR loci were identified using the SSRHunter 1.3 software (parameter settings: dinucleotide repeats ≥ 6 times, trinucleotide repeats ≥ 4 times). A higher threshold of repeat counts can effectively filter out a large number of randomly occurring short repeat fragments in the genome, reducing false positives and ensuring that SSRs have high polymorphism, stable amplification and practicality. Primers for SSR loci with more than two polymorphisms were designed using primer3 v2.3.6 with the target amplification product size spanning from 100 to 400 bp (Untergasser et al., 2012).

#### SSR primer screening and PCR amplification for *P. zhennan*

A total of 16 *Phoebe zhennan* samples from ten different provenances in Guizhou (SQ (two), SN (two), DJ (two), CS (one), XS (one), JK (two), WC (two), ZG (one), MT (two), DZ (one); Table S1) were used as experimental materials. Genomic DNA was extracted from leaf tissue using a modified cetyltrimethylammonium bromide (CTAB) method. A total of 108 SSR primer pairs, designed to meet criteria including length (18–27 bp), Tm (≤ 65 °C), and absence of polypurine/polypyrimidine tracts, were synthesized by Shanghai Bioengineering Company. Excluding single nucleotides and compound repeat motifs, the requirement for controlling the distribution density of SSRs demands that each single read contain only one SSR. The extracted genomic DNA served as the template for PCR amplification. Reactions (10 µL final volume) contained 0.2 µL each of forward and reverse primer, 1 µL *P. zhennan* DNA template, 5 µL Thermo Scientific DreamTaq Green PCR Master Mix (2X), and 3.6 µL ddH_2_O. Amplification was performed under the following conditions: initial denaturation at 95 °C for 3 min; 35 cycles of denaturation at 94 °C for 30 s, annealing for 30 s, extension at 72 °C for 50 s; final extension at 72 °C for 10 min; hold at 4 °C. Preliminary screening of all 108 primer pairs was conducted *via* 1% agarose gel electrophoresis. Based on amplification efficiency and specificity, 33 pairs were retained for further analysis; primers exhibiting low amplification efficiency or non-specific amplification were excluded. PCR amplification using the 33 pairs was repeated, and amplicons were analyzed by 2% agarose gel electrophoresis. Subsequently, SSR-PCR products were subjected to capillary electrophoresis (Agilent 2100 Expert Software, vB.02.03.SI301; Fig. S1) to further assess primer performance, focusing on band stability and polymorphism level. Finally, based on the polymorphism level measured by Polymorphism Information Content (PIC) and consistent amplification of highly polymorphic bands, 20 optimal primer pairs were selected for subsequent studies (Table S2).

#### Data analysis

The results of capillary electrophoresis were scored as 0 or 1, and GD parameters were calculated based on manual band reading. GD indices, including the number of alleles (Na), the effective number of alleles (Ne), Shannon information index (I), genetic differentiation coefficient (Gst), and gene flow (Nm), were computed using GenAlEx 6.51 software (Peakall & Smouse, 2012). Nei’s GD index (H) was determined by Power Marker 3.25 software (Liu & Muse, 2005). UMPGA clustering of populations was performed based on Nei’s genetic distances, and the resulting cluster tree was visualized through the iTOL online website (https://itol.embl.de/) (Letunić & Bork, 2024). Molecular ANOVA was conducted using Arlequin 3.5.2.2 (Excoffier & Lischer, 2010). Population genetic arrangement was examined using Structure 2.3.4 software to categorize the populations (Pritchard, Stephens & Donnelly, 2000). The results folder was then compressed and submitted to Structure Harvester (0.6.94) (Earl & vonHoldt, 2011).

## Results

### Characterization of SSR loci

**Figure 1:**
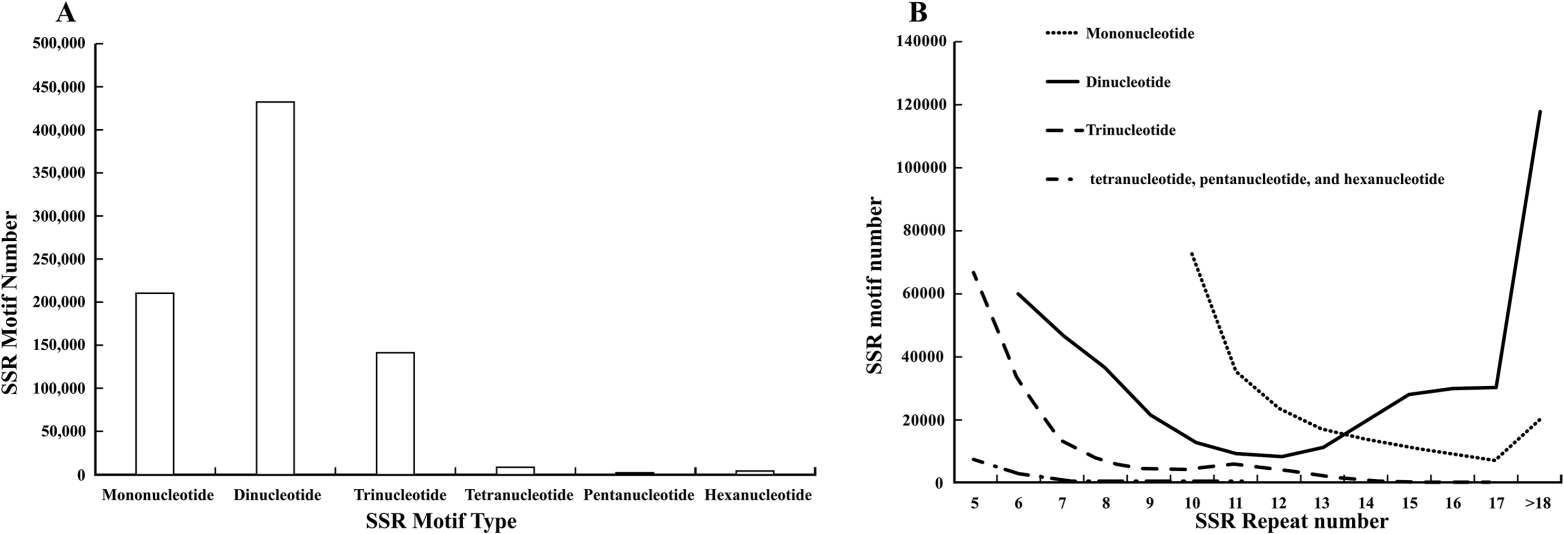
Analysis of SSRs site characteristics. (A) Graphical breakdown of SSR motif types; (B) distribution of repeat counts.

Characterization of the SSR sites revealed (Fig. 1A) that dinucleotide SSRs comprised the highest percentage, with 431,326 occurrences, accounting for 54.31%. This was followed by mononucleotide SSRs and trinucleotide SSRs, with 209,961 and 141,218 respectively, representing 26.44% and 17.78%. The occurrences of tetranucleotide, pentanucleotide, and hexanucleotide SSRs were lower, collectively accounting for 1.47%. Among these, the most common mono-nucleotide were of the A/T type, constituting 98.81%, while the AG/CT type of dinucleotide SSRs exhibited the highest frequency of occurrence, at 84.73%. The dominance of single-nucleotide A/T repeats and the high abundance of dinucleotide AG/CT repeats are “functional adaptive features” formed by the genome during long-term evolution: the former participates in gene expression and evolutionary adaptation by regulating chromatin structure and providing high variability, while the latter is more focused on the fine regulation of RNA processing and chromatin regulation. These “sequence preferences” of repeats are closely related to their functions and are important clues for understanding the relationship between genome structure and function. Most trinucleotide SSRs were of the AAG/CTT type, representing 80.08%. The Mono-nucleotide predominantly exhibited repeat numbers ranging from 10 to 15, while dinucleotide SSRs were centered within the six and 10 repeat range. Trinucleotide SSR repetitions were primarily concentrated between five and seven repeats, while tetranucleotide, pentanucleotide, and hexanucleotide SSRs were predominantly limited to five repetitions. Further analysis indicated that as the number of repeats in the repeat motifs increased, the percentages in each category decreased, and the number of SSR loci gradually reduced (Fig. 1B).

### Polymorphism analysis of the SSR markers

A total of 218 alleles were identified across 16 individuals using the 20 selected SSR primers. The number of alleles per locus ranged from seven to 15, with an average of 10.9. The observed number of effective Ne varied from 1.693 to 2.667, with a mean of 2.260. Shannon’s information index (I) ranged from 0.472 to 0.970, with a mean of 0.763. The mean values of observed heterozygosity (Ho) and expected heterozygosity (He) were 0.705 and 0.487. This might imply heterozygote advantage or population structure. Nei’s GD index (H) ranged from 0.801 to 0.910, with a mean of 0.872. The PIC of the SSR loci ranged from 0.777 to 0.903, with an average of 0.859. These results indicated that the 20 SSR loci were highly polymorphic and could be effectively used for assessing the GD of *P. zhennan* (Table 3).

**Table 3:** SSR primer screening results.

Prime	Allele	Number of effective alleles (Ne)	Shannon Information Index (I)	Observational heterozygosity (Ho)	Expected heterozygosity (He)	Nei’s GD index (H)	Polymorphic information content (PIC)
SSR-1	7	2.360	0.784	0.650	0.475	0.830	0.808
SSR-2	9	2.127	0.749	0.600	0.488	0.816	0.793
SSR-3	12	2.127	0.645	0.400	0.400	0.896	0.888
SSR-4	11	1.867	0.589	0.450	0.388	0.881	0.869
SSR-5	10	2.127	0.645	0.550	0.400	0.865	0.851
SSR-6	9	2.133	0.624	0.400	0.375	0.854	0.837
SSR-7	10	1.967	0.624	0.300	0.400	0.857	0.842
SSR-8	8	1.693	0.472	0.350	0.313	0.826	0.804
SSR-9	8	1.967	0.624	0.350	0.400	0.844	0.825
SSR-10	8	2.433	0.832	0.700	0.525	0.801	0.777
SSR-11	12	2.033	0.693	0.850	0.475	0.881	0.869
SSR-12	13	2.400	0.866	0.950	0.563	0.906	0.899
SSR-13	15	2.600	0.936	0.900	0.588	0.910	0.903
SSR-14	12	2.600	0.936	0.850	0.588	0.885	0.874
SSR-15	13	2.567	0.866	0.900	0.538	0.910	0.903
SSR-16	11	2.467	0.866	1.000	0.563	0.879	0.867
SSR-17	12	2.267	0.832	1.000	0.550	0.893	0.883
SSR-18	14	2.333	0.832	0.950	0.550	0.908	0.901
SSR-19	11	2.667	0.970	0.950	0.600	0.889	0.878
SSR-20	13	2.467	0.866	1.000	0.563	0.906	0.899
Mean	10.9	2.260	0.763	0.705	0.487	0.872	0.858

### GD analysis of Guizhou population

The analysis results revealed that, in Guizhou *P. zhennan* populations, the Na value ranged from 3.850 to 8.150, with a mean of 5.581. The highest Na value was observed in the SN population, while the minimum values were found in the JH and MT populations. The Ne value ranged from 3.313 to 6.402, and the I value varied from 1.196 to 1.928, with the maximum and minimum values of Ne and I observed in the SN and MT populations, respectively. The Ho values ranged from 0.406 to 0.823, with the maximum value in the JK and the minimum in the DZ. The He values ranged from 0.654 to 0.831, with the maximum in the SN and the minimum in the MT. In addition, the Ho values for the *P. zhennan* populations were smaller than the He values, except in DJ, JH, and MT, suggesting the possibility of inbreeding within these populations. The variation in Nei’s GD Index (H) ranged from 0.661 to 0.832, with the highest value in the SN population and the lowest in the MT population. Overall, these results suggested that the SN population exhibited higher GD, while the MT population had lower GD. Moreover, when compared with other provinces, the differences in Na, Ho, He, *etc.* were statistically significant (Table 4).

**Table 4:** Genetic diversity analysis of population.

Species		Na	Ne	I	Ho	He	H
*Phoebe zhennan*	CS	6.250	4.832	1.621	0.727	0.750	0.751
	DJ	5.000	4.094	1.456	0.798	0.732	0.737
	DZ	4.550	3.784	1.335	0.406	0.680	0.701
	FG	5.050	3.854	1.398	0.650	0.711	0.714
	JH	3.850	3.423	1.223	0.750	0.663	0.664
	JK	6.100	4.668	1.608	0.823	0.765	0.773
	MT	3.850	3.313	1.196	0.675	0.654	0.661
	SN	8.150	6.402	1.928	0.788	0.831	0.832
	SQ	7.750	5.292	1.807	0.738	0.799	0.811
	TJ	5.500	4.597	1.540	0.740	0.744	0.744
	TZ	4.200	3.315	1.273	0.577	0.676	0.685
	WC	5.050	4.339	1.515	0.668	0.758	0.762
	XS	4.800	4.227	1.442	0.725	0.731	0.736
	YQ	8.000	6.015	1.856	0.705	0.807	0.809
	ZA	6.350	5.152	1.691	0.732	0.784	0.785
	GL	5.900	4.513	1.553	0.637	0.741	0.763
	BJ	6.200	4.507	1.579	0.652	0.733	0.739
	TN	6.800	4.536	1.650	0.725	0.764	0.779
	ZG	4.700	3.992	1.400	0.590	0.713	0.713
	HJ	6.500	5.067	1.699	0.690	0.789	0.793
*Phoebe bournei* ^*^	MN	8.500	6.087	1.917	0.795	0.821	0.821
*Phoebe sheareri* ^*^	ZIN	8.000	5.704	1.863	0.790	0.811	0.816
*Phoebe chekiangensis* ^*^	ZJN	6.800	4.578	1.648	0.802	0.759	0.767
*Lindera megaphylla* ^#^	YH	4.850	3.872	1.395	0.720	0.710	0.719

**Notes.**

* other species of the *Phoebe* genus.

# other species of the *Lindera* genus.

When comparing the GD parameters of *P. zhennan* from Sichuan, Chongqing, and Hunan to those in Guizhou, the BJ, GL, ZG, and TN populations showed intermediate GD, lower than the SN population but higher than the MT population. The Nei’s GD indices for BJ, GL, ZG, and TN were all greater than 0.7, suggesting that these populations maintain high GD. Additionally, the Ho values for BJ, GL, ZG, and TN were all smaller than their respective He values, indicating a certain degree of inbreeding in these populations. A comparison of the GD indices between the collected *P. zhennan* from Hejiang County, Sichuan Province, and other species such as *P. bournei*, *P. chekiangensis*, and *P. sheareri* showed that the Shannon information index for the *P. bournei* population was the highest, at 1.917. The Ho value for *P. chekiangensis* reached a maximum of 0.802, while the maximum He value for *P. bournei* was 0.821. The Nei’s GD indices (H) ranked from high to low as *P. bournei*, *P. sheareri*, *P. zhennan*, and *P. chekiangensis*, with the combined analysis confirming that all four *Phoebe* species exhibited high GD.

### Analysis of genetic differentiation of Guizhou *P. zhennan* populations

The genetic differentiation of Guizhou *P. zhennan* populations was analyzed based on 20 SSR loci (Table 5). Fis represents the inbreeding coefficient within a population, and Fit represents the inbreeding coefficient between populations. The Fis values for the 20 SSR loci ranged from −0.323 to 0.478, with a mean value of 0.059. The Fit values for the 20 SSR loci ranged from −0.089 to 0.628, with a mean value of 0.218, suggesting the presence of some degree of inbreeding between populations. First, the coefficient of differentiation between populations ranged from 0.114 to 0.288, with a mean value of 0.175, indicating significant genetic differentiation among populations. The mean Fst value suggests a relatively high level of genetic differentiation. Nm, which measures gene flow, ranged from 0.619 to 1.941, with a mean value of 1.263. Since Nm >1, this indicates frequent gene flow between populations, with alleles from each locus being widely distributed across populations.

**Table 5:** Genetic differentiation analysis of the *P. zhennan* population.

Locus	Fis	Fit	Fst	Nm
SSR-1	0.049	0.158	0.114	1.941
SSR-2	0.212	0.383	0.217	0.900
SSR-3	0.441	0.524	0.148	1.444
SSR-4	0.290	0.433	0.202	0.985
SSR-5	0.167	0.382	0.258	0.718
SSR-6	0.478	0.628	0.288	0.619
SSR-7	0.341	0.464	0.186	1.097
SSR-8	0.238	0.390	0.199	1.007
SSR-9	0.255	0.421	0.223	0.871
SSR-10	0.367	0.447	0.125	1.745
SSR-11	−0.123	0.051	0.156	1.357
SSR-12	−0.137	0.030	0.146	1.457
SSR-13	0.165	0.289	0.148	1.437
SSR-14	−0.101	0.080	0.164	1.273
SSR-15	−0.228	−0.054	0.141	1.518
SSR-16	−0.323	−0.089	0.177	1.162
SSR-17	−0.223	−0.024	0.163	1.287
SSR-18	−0.228	−0.045	0.149	1.433
SSR-19	−0.267	−0.063	0.161	1.300
SSR-20	−0.196	−0.044	0.128	1.711
Mean	0.059	0.218	0.175	1.263

An analysis of molecular variance (AMOVA) (10,000 permutations) of the 20 loci in Guizhou *P. zhennan* populations was conducted using Arlequin software (Table 6). A total of 218 alleles were identified across 16 individuals using the 20 selected SSR primers. This implies that the majority of genetic variation in Guizhou *P. zhennan* is found within individual populations.

**Table 6:** Analysis of molecular variance of the *P. zhennan* population.

Source of mutation	Degrees of freedom	Square sum	Variance component	Variance component ratio
Intergroup	15	260.086	0.76630	9.43%
Intragroup	194	1,428.061	7.36114	90.57%
Total	209	1,688.148	8.12,744	
Fst:0.09429				

### Analysis of the genetic structure of populations

**Figure 2:**
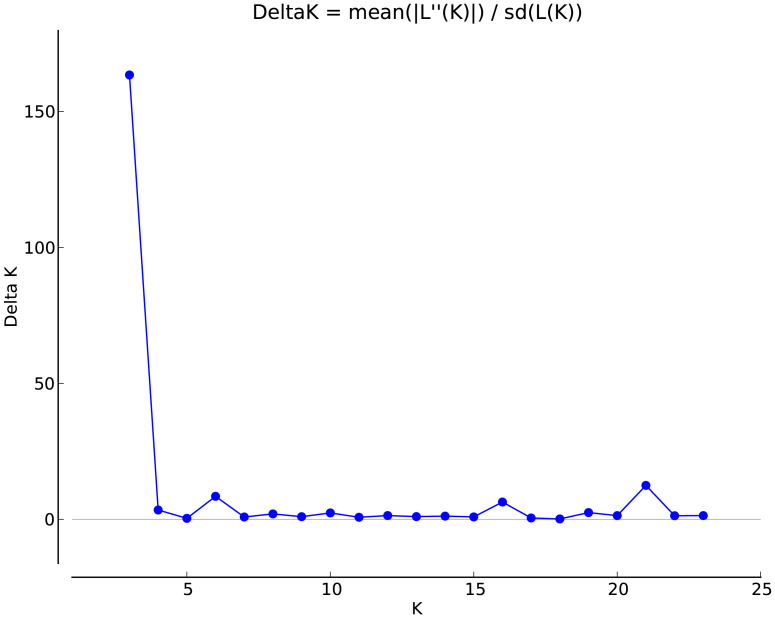
Selection of optimal *K* value.

**Figure 3:**
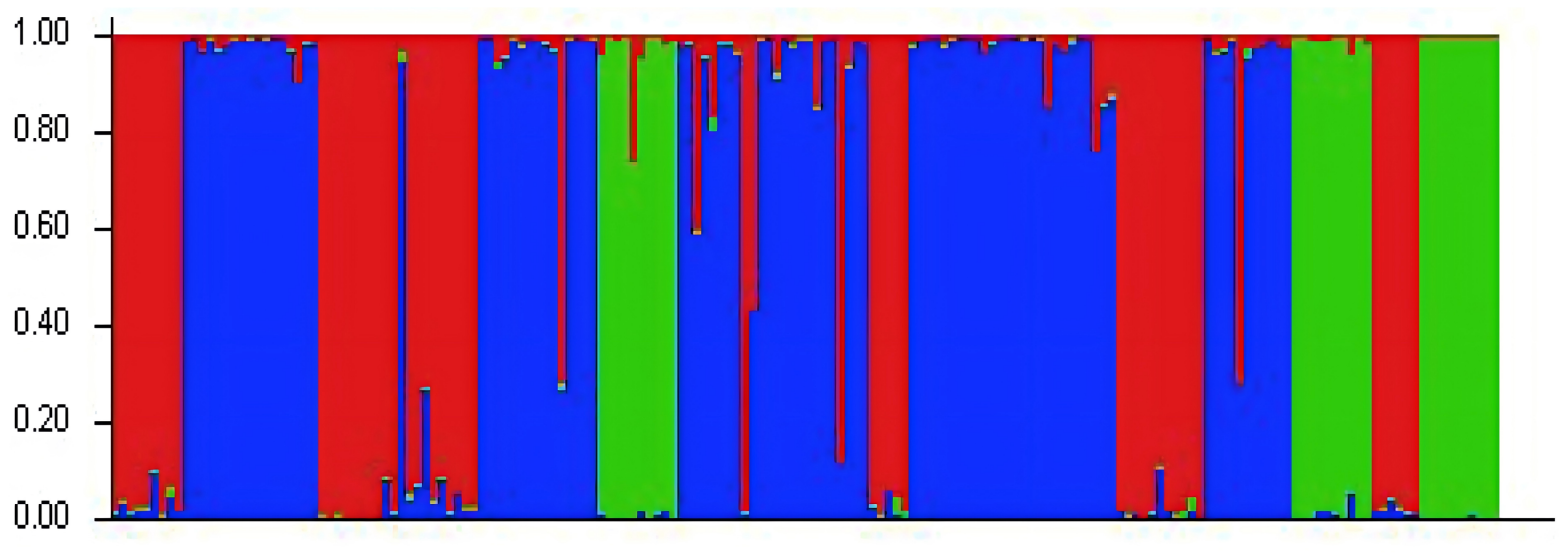
Genetic structure analysis results.

The genetic structure of the populations was analyzed using Structure software (Fig. 2). The highest ΔK value was observed when *K* = 3, indicating that the 24 populations could be classified into three subgroups, which likely originated from three distinct homologous gene pools. The classifications are represented by red, green, and blue colors (Fig. 3). The classification was based on the *Q* value, where *Q* ≥ 0.6 indicates a relatively homogeneous genealogy, allowing the individuals to be clustered into a single group (Table S3). The first group (red) includes 54 individuals from 13 populations, accounting for 31.03% of the total. This group consists of all individuals from the BJ, FG, GL, JH, TJ, YQ, and YH groups, and some individuals from the CS (two), JK (one), HJ (one), SN (one), SQ (one), and ZA (one) groups. The second group (green) includes all individuals from three populations: ZIN (*P. sheareri*), ZJN (*P. chekiangensis*), and MN (*P. bournei*), accounting for 17.24% of the total. The third group (blue) consists of 88 individuals from 14 populations, accounting for 50.57% of the total. This group includes all individuals from the DJ, DZ, TN, TZ, WC, XS, and ZG groups, as well as some individuals from the CS (five), GL (one), JK (seven), HJ (seven), MT (three), SN (seven), SQ (10), and ZA (five) groups. In addition, MT20-03 and SN20-11 exhibit *Q* < 0.6, suggesting a more complex genealogy, and these two individuals were categorized into a fourth group.

### Cluster analysis and principal component analysis of populations

**Figure 4:**
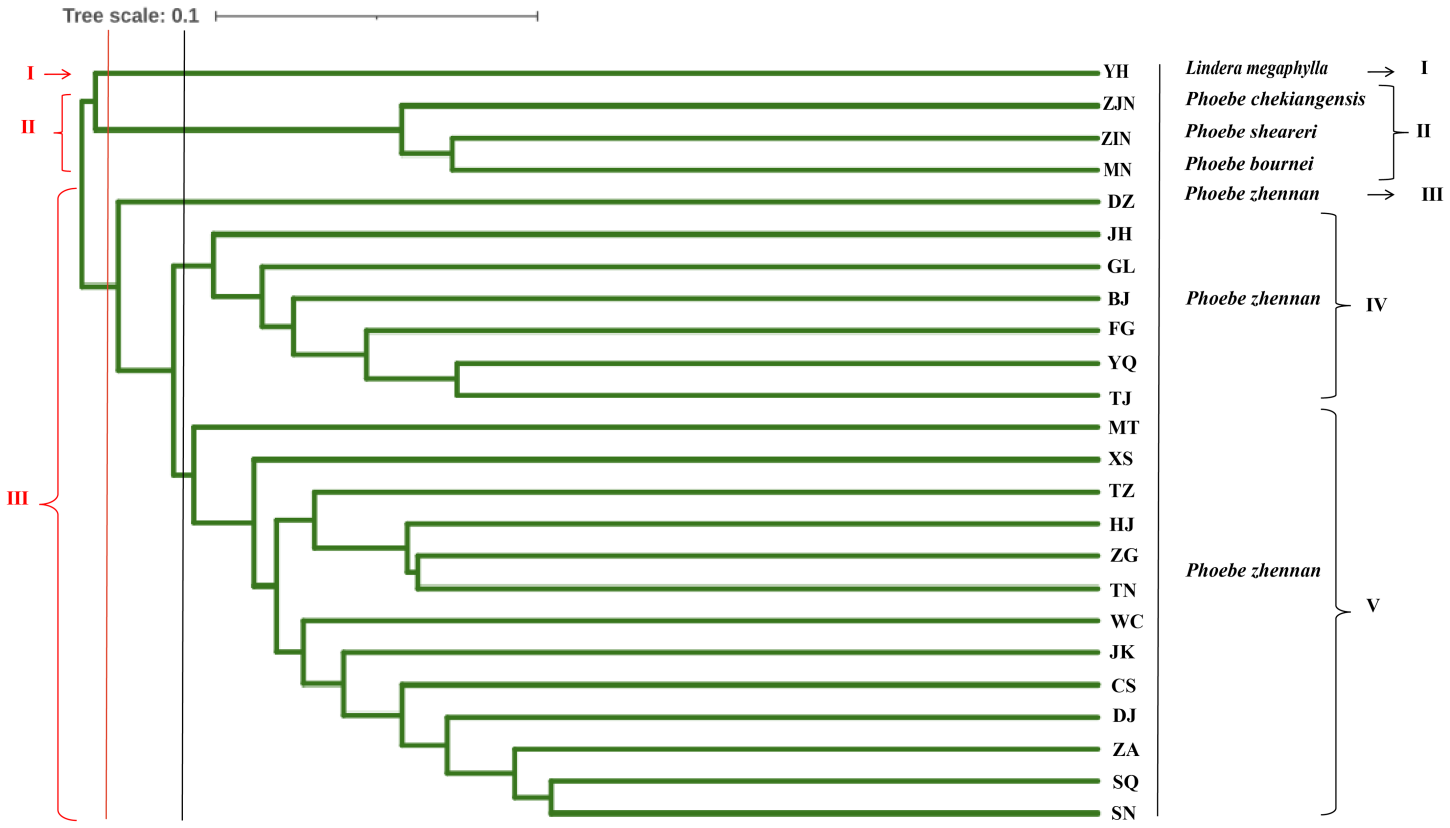
Genetic diversity parameters in 20 populations of *P. zhennan*, and GD comparison with four other related species.

UPGMA cluster analysis was conducted based on the Nei’s genetic distance between populations (Fig. 4). When the 24 populations were clustered into three groups, the first group was the *L. megaphylla* population, the second group included the *P. bournei, P. sheareri*, and *P. chekiangensis* populations, and the third group included all *P. zhennan* populations. When the 24 populations were clustered into five groups, the first and second groups were the same as in the three-group classification. The third group included only the DZ *Phoebe* population, the fourth group was the JH, GL, BJ, FG, YQ, and TJ *Phoebe* populations, and the fifth group was composed by the remaining populations of *P. zhennan.* Comprehensive analysis shows that this study can well distinguish *P. bournei* from *Phoebe*, and it is believed that *P. bournei* has a closer kinship with *P. chekiangensis* and *P. sheareri* belong to the same branch, while the rest of the populations are clustered into one branch. Among them, the HJ, ZG, TN, and TZ *Phoebe* populations belong to the same branch and have a closer kinship. The sample population collected from Yanhe County, Guizhou Province, was clustered into a separate group, which might be another species of the *Phoebe* genus.

**Figure 5:**
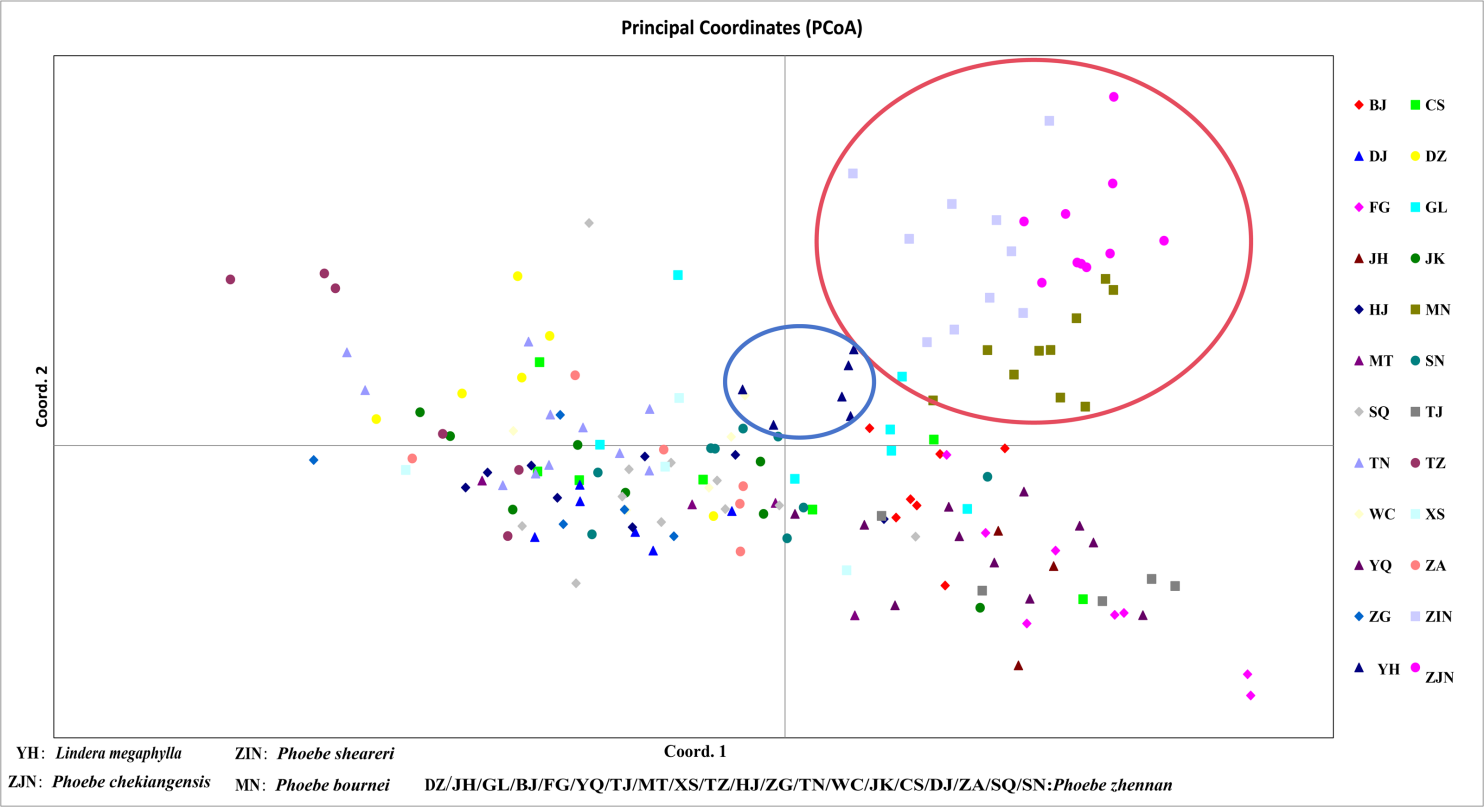
Principal component analysis.

In a principal component analysis of 174 samples based on Nei’s genetic distance (Fig. 5), all individuals from the three groups—*P. bournei*, *P. sheareri*, and *P. chekiangensis*—were clustered into one group in the upper right corner (red), with no overlap between them. The YH individuals formed a distinct group on their own (blue), while the remaining *P. zhennan* groups were distributed in a cross pattern, in agreement with the findings of the cluster analysis.

## Discussion

The morphological differences among species of the genus *Phoebe* are relatively small and they are widely distributed. Despite the use of traditional morphological classification and currently developed EST and transcriptome molecular markers, resolving interspecific relationships and conducting accurate species identification within the genus *Phoebe* remains challenging. In particular, *P. zhennan* and *P. bournei* are often considered taxonomically controversial species due to their minor differences in leaf morphological traits, which are further influenced by environmental factors. Li et al. (2018) failed to distinguish *P. zhennan* from *P. bournei* using ISSR markers and suggested merging the two species. In contrast, Ding et al. (2018) integrated morphology data with RAD-seq sequencing to distinguish *P. zhennan* from *P. bournei* through SNP detection. Compared to SNP detection technology, SSR markers, as a second-generation molecular marker technology, offer low-cost, accurate detection, and were developed and applied earlier. For example, Wang (2016) developed SSR molecular markers based on the whole genome of *Camphora officinarum* and demonstrated the applicability of 27 pairs of markers across the genera *Phoebe* and *Machilus*. An AMOVA of the 20 loci in Guizhou *P. zhennan* populations. Miao et al. (2023) used SSR marker technology to confirm that *Pinus yunnanensis* is a typical outcrossing species and maintains medium to high GD.

This study constructed an SSR-enriched genomic library of the *P. zhennan* genome based on the magnetic bead enrichment method and developed SSR loci of the *P. zhennan* genome, which enriched the molecular marker resources of *P. zhennan* and its related species. A total of 794,128 SSR loci were identified, exceeding the number obtained from the transcriptome data. These SSRs exhibited higher polymorphism levels, as indicated by polymorphism information content (PIC) values, compared to traditional EST-SSRs, while also offering lower development costs than single-nucleotide polymorphism (SNP) technologies. The SSR markers developed for *P. zhennan* also demonstrated a high transferability rate to related species (Zhang et al., 2019), thereby supporting comparative genomics research across species within the *Phoebe* genus. The extent of allele sharing among species may be further used to reconstruct phylogenetic relationships in the genus (Paliwal et al., 2022). Among the SSR loci identified in this study, mononucleotide and dinucleotide repeats accounted for 80.75%, indicating a high evolutionary level for *P. zhennan*. Genetic diversity is an important index to measure whether a species can develop stably, and molecular marker technologies enable the assessment of genetic variation at the gene level, independent of environmental influences (Bhatia & Banga, 2022). The 20 pairs of polymorphic primers screened in this study can be applied in subsequent genetic diversity analysis and molecular marker-assisted breeding of *P. zhennan*. High genetic diversity individuals (He > 0.6) can be selected as core breeding parents through MAS (Li et al., 2022), while populations with fewer than three alleles may be identified as bottlenecked, informing *ex-situ* conservation and reintroduction strategies when combined with ecological data (Sharma et al., 2019). This approach helps prevent the loss of adaptability due to inbreeding depression.

*P. zhennan* was studied using 20 SSR in this work. The observed Ho value of 0.701 was lower than the expected He value of 0.737, indicating a certain degree of inbreeding within the Guizhou *P. zhennan* population. The GD levels of the Guizhou population, in descending order, were as follows: SN > SQ > YQ > ZA > JK >  WC > CS > TJ > DJ > XS > FG > DZ > TZ > JH > MT. When compared to *P. chekiangensis* (*H* = 0.3206) and other endangered woody plants (*Liriodendron chinense, H* = 0.740; *Senegalia pennata*, *H* = 0.573; *Dalbergia nigra*, *H* = 0.740) (Li, 2013; Gao & Li, 2008; Buzatti et al., 2012), the GD of Guizhou *P. zhennan* populations (*H* = 0.743) was found to be relatively high. This indicates that even though the Guizhou *P. zhennan* population is fragmented, it may maintain high GD due to its earlier origin, longer lifespan, and the accumulation of genetic variation. Among the populations, MT, JH, TZ, and DZ exhibited GD indices (H) between 0.6 and 0.7, which are lower compared to the rest of the Guizhou populations. These populations require special conservation attention to maintain GD. On the other hand, the GD indices (H) of FG, XS, DJ, TJ, CS, WC, JK, and ZA ranged between 0.7 and 0.8, suggesting they are relatively stable and can be conserved under the current protection measures. The populations of SN, SQ, and YQ, with GD indices (H) greater than 0.8, demonstrate high GD and should be prioritized for protection. These populations could also serve as sources for screening superior *P. zhennan* plants. Furthermore, a comparison of the GD of the Guizhou *P. zhennan* population with those from Sichuan, Hunan, and Chongqing revealed that the populations in these regions also possess high GD. In summary, despite the significant decline of *P. zhennan* resources in the past due to natural disasters and anthropogenic factors, the overall GD of the *P. zhennan* population remains high.

The spatial heterogeneity of genetic diversity in *P. zhennan* reflects not only the historical dynamics of population size, but also the influence of the altitude gradient and the degree of habitat fragmentation. In high-altitude areas (*e.g.*, SN, SQ, and YQ), relatively stable climatic conditions and reduced human disturbance may have allowed for the preservation of more intact population structures, promoting the accumulation of adaptive genetic variation (Geng et al., 2017). In contrast, low-altitude regions (*e.g.*, MT and JH) are subject to more intense anthropogenic pressures, such as deforestation and agricultural expansion, which can lead to population decline, increased inbreeding, and consequently, a decrease in genetic diversity (Zhao, Yin & Gong, 2022). Furthermore, in regions with lower diversity, such as MT and DZ, habitat fragmentation may cause population isolation, accelerating genetic drift and allele loss (Brunke et al., 2020). Conversely, in areas with higher diversity (*e.g.*, SN and SQ), larger effective population sizes are likely maintained, which can mitigate the effects of inbreeding depression and genetic drift (Fraser, 2017), thereby enhancing adaptive potential in response to environmental stressors such as drought or pest outbreaks. To support long-term genetic health in *P. zhennan*, measures such as restoring forest corridors and limiting human disturbance in low-altitude areas should be considered. These efforts may promote gene flow among populations and help maintain evolutionary resilience (Luna et al., 2023). In summary, although various natural disasters and human factors in the past have led to a sharp decline in *P. zhennan* resources, the overall genetic diversity level of the current *P. zhennan* populations remains relatively high, indicating that the genetics of *P. zhennan* are relatively stable. However, human intervention is still needed for the protection and breeding of *P. zhennan*. Population genetic differentiation is an indicator that reflects the genetic structure of a population. The main factors influencing genetic differentiation include genetic drift, gene flow, and evolutionary history. Previous studies have shown that gene flow between populations can effectively prevent genetic differentiation due to genetic drift when the value of Nm >1 (Zacharias et al., 2021). The results of genetic differentiation in this study indicated that the majority of genetic differentiation occurred within populations, with 82.5% of the genetic variation observed within populations and 17.5% between populations. Specifically, 9.43% of the total genetic variation was attributed to differences between populations, while 90.57% was within populations. This suggests that the Guizhou *P. zhennan* populations exhibit a high degree of geographic coherence, with minimal genetic differentiation resulting from geographic isolation. The gene flow (Nm = 1.354) was greater than 1, indicating that there is sufficient gene exchange between the Guizhou *P. zhennan* populations to prevent genetic differentiation due to genetic drift. This also suggests that wild *P. zhennan* populations can remain stable in the absence of anthropogenic. Additionally, the *P. zhennan* populations in this study mainly came from mountainous regions, while the dispersal of *Lauraceae* seeds relies largely on birds, gravity, or human activities, which may further contribute to genetic exchange among Guizhou *P. zhennan* populations.

A total of 174 individuals from 24 populations were assessed for their genetic structure. The results revealed that the populations could be roughly classified into three groups: the first group, the second group, and the third group, which accounted for 31.03%, 17.24%, and 50.57% of the total populations, respectively. Two individuals, MT20-03 and SN20-11, showed a *Q*-value of less than 0.6 and exhibited more complex genetic backgrounds, so they were categorized into a fourth group. UPGMA clustering analysis based on Nei’s genetic distance revealed that samples collected from HJ were clustered with the ZG, TN, and TZ populations. This suggests that the ZG, TN, and TZ populations were more closely related to the Hejiang samples from Luzhou. Additionally, *P. zhennan* samples from Gulin County, Sichuan Province, were clustered with the BJ, FG, YQ, and TJ groups, indicating that these groups of *P. zhennan* were more closely related to one another. Both cluster and principal component analyses grouped the YH population separately, indicating that YH was genetically more distant from the other groups. During the sample collection process, based on the local people’s description, this species was identified as “*P. zhennan*”, and was confirmed as *L. megaphylla* by Professor Mingtai from Guizhou University. However, molecular marker analysis revealed that the genetic distance between this population sample and *P. zhennan, P. bournei, P. chekiangensis* and *P. sheareri* was relatively large, thereby providing molecular-level evidence supporting the accurate identification of Guizhou *P. zhennan* resources. The genetic distance between the Guizhou *P. zhennan* populations was not found to correlate with geographical distance. This finding suggests that factors other than geography may influence the genetic structure of the populations, and the underlying reasons for this need further investigation at a deeper level (Hamrick & Godt, 1996). This study provides abundant SSR resources for the genetic diversity analysis and molecular identification within the *Phoebe* genus. The results indicate that SSR markers of *P. zhennan* are more effective and reliable than previously used marker systems such as ISSR, SRAP and AFLP markers. The basic genetic data of the wild population of *P. zhennan* in Guizhou obtained in this study provide a clear framework for the establishment of the endangered species *P. zhennan* in Guizhou. In particular, the SN population with higher genetic diversity should be prioritized for both *ex-situ* and *in-situ* conservation strategies. These materials can be used for the selection of superior individuals and artificial cultivation. However, several limitations remain. The developed SSR markers are based on simplified genome sequencing and cannot cover the repetitive regions of the whole genome, which may miss important variations in regulatory regions (Zhou et al., 2021). Moreover, the co-occurrence of *Phoebe fortunei* and *P. zhennan* in certain habitats raises the possibility of gene introgression, which requires further verification through high-resolution SNP marker analysis (Zhang et al., 2024). In addition, during the collection of *P. zhennan* samples, it was observed that the *P. zhennan* at the collection site were seriously infested with pests and diseases, and were not adequately treated. Meanwhile, *P. bournei* in Guizhou was found to be mixed with *P. zhennan*, and accurate data on *P. zhennan* populations were not properly recorded during the listing process, which has caused some challenges in the conservation of *P. zhennan* species. It is recommended to strengthen the prevention and control of pests and diseases affecting *P. zhennan*, conduct a comprehensive census of *P. zhennan* resources in Guizhou, and rectify the listing information.

## Conclusions

In this study, *P. zhennan* genomic SSR molecular markers were developed based on the sequencing of genomic SSR-enriched libraries. Analysis using 20 highly polymorphic SSR loci demonstrated that all Guizhou *P. zhennan* populations exhibited high GD, with the SN *P. zhennan* population showing the highest GD, while the MT population exhibited lower diversity. The genetic differentiation within the Guizhou *P. zhennan* populations was found to be primarily intra-population. Cluster analysis revealed that the *P. zhennan* populations from ZG, TN, and TZ were more closely related. Furthermore, the polymorphic primers identified in this study effectively distinguished *P. zhennan* from *P. bournei*, *P. sheareri*, and *P. chekiangensis*.

##  Supplemental Information

10.7717/peerj.20434/supp-1 Supplemental Information 1 Partial results of capillary electrophoresis for PCR primers

10.7717/peerj.20434/supp-2 Supplemental Information 2 Information on the collection sites of *P. zhennan* samples

10.7717/peerj.20434/supp-3 Supplemental Information 3 20 pairs of *P. zhennan* SSR primers

10.7717/peerj.20434/supp-4 Supplemental Information 4 The *Q* values of 24 populations of *P. zhennan*

## Additional Information and Declarations

### Data Availability

The following information was supplied regarding data availability: The raw sequence reads are available at NCBI: PRJNA1224921.
