# Supplementary material for: Development and characterization of SSR markers in Phoebe zhennan
Source: PeerJ. 2025 Dec 15;13:e20434. doi: 10.7717/peerj.20434 (PMC12713558; doi:10.7717/peerj.20434)
Supplement: Supplemental Information 1 [file peerj-13-20434-s001.docx]

| Sampling site | Sample Name | Accession | Amount |
| --- | --- | --- | --- |
| Shiqian County, Guizhou Province | SQ | SQ20-07、SQ20-17 | 2 |
| Sinan County, Guizhou Province | SN | SN20-11、SN20-17 | 2 |
| Dejiang County, Guizhou Province | DJ | DJ20-03、DJ20-04 | 2 |
| Wuchuan County, GuizhouProvince | WC | WC20-01、WC20-04 | 2 |
| Meitan County, Guizhou Province | MT | MT20-03、MT20-04 | 2 |
| Jiangkou County, Guizhou Province | JK | JK20-02、JK20-08 | 2 |
| Xishui County, Guizhou Province | XS | XS20-01 | 1 |
| Chishui City, Guizhou Province | CS | CS20-01 | 1 |
| Zhengan County, Guizhou Province | ZA | ZA20-03 | 1 |
| Daozhen County, Guizhou Province | DZ | DZ23-05 | 1 |
| Total |  |  | 16 |
