# Supplementary material for: Development and characterization of SSR markers in Phoebe zhennan
Source: PeerJ. 2025 Dec 15;13:e20434. doi: 10.7717/peerj.20434 (PMC12713558; doi:10.7717/peerj.20434)
Supplement: Supplemental Information 2 [file peerj-13-20434-s002.docx]

| Primers | Primer sequence | Primers | Primer sequence |
| --- | --- | --- | --- |
| SSR-1 | F: GAACTTCTGGAGTGCACGG  R: TTGGCCATCAACACTAAGACC | SSR-11 | F: TCTATCAACATGAGCAGGCTGT  R: CCTATCAACATGCACCAACTG |
| SSR-2 | F: GCCTGAAGGATTGGTGTTGT  R: GCTCTTCCTGCATTCCTACG | SSR-12 | F: ATTCATGTACGTCCGGCTTC  R: TGTTCGCACAAGAGATGGAG |
| SSR-3 | F: GGATGGCCTGAAGCATGTAT  R: TCCGAGCCGTATACTTCCAT | SSR-13 | F: TGAGGAAGAAGGAGTGCAGAG  R: CGATCACCAATTGCATTCAC |
| SSR-4 | F: CCACTCGTGTGCATGATTCT  R: TCTTCCACAACTACCTGTCGTC | SSR-14 | F: TCTGATGCTATGCCTGAACG  R: TAATTGCATTGCTCGTCTGC |
| SSR-5 | F: GCGAGATGTTGTGTGCACTT  R: CCTCGAATAGCATCCTCAGC | SSR-15 | F: CCTACACTATGGTGGCCTCTG  R: ATGCACTTACCTCCATTGGC |
| SSR-6 | F: AGCCTCAACTGTCATTGCCT  R: ATTACGAGAGCGAGAGCGAG | SSR-16 | F: CATTGAAGATTGCACCTTGG  R: CCATTGCCATAACAGTGGTG |
| SSR-7 | F: AAGCCTTCCTACAAGCCTCC  R: AAGAATGTGAAGCGCTGGTT | SSR-17 | F: CCTATTCGTTCAGTGATTGGC  R: ACTACTGCGTGATCCTGCCT |
| SSR-8 | F: GCAAGCAAGGTTCTGACCTC  R: ACATTAATGGTAGCGGCTGG | SSR-18 | F: GGAGATGAGATGGCCTATGG  R: CGGAGTGGTGGAAGAGAGTC |
| SSR-9 | F: ATGGTGAAGGAAGGATGCAC  R: GACTTAAGCCAACGCATTGAT | SSR-19 | F: TCCTACAGATGTGCCAGTGC  R: TGACATTGATGATTCCGAGG |
| SSR-10 | F: TCTTGCAGATTCCGTGTGAG  R: CTTCACTTCCTCTCCAACCG | SSR-20 | F: AGTTGGTTGTGGCACTTATGG  R: ATGTATTGGCGCTTGGATTC |
