# Supplementary material for: Development and characterization of SSR markers in Phoebe zhennan
Source: PeerJ. 2025 Dec 15;13:e20434. doi: 10.7717/peerj.20434 (PMC12713558; doi:10.7717/peerj.20434)
Supplement: Supplemental Information 3 [file peerj-13-20434-s003.docx]

| Number | Q1 | Q2 | Q3 | Number | Q1 | Q2 | Q3 |
| --- | --- | --- | --- | --- | --- | --- | --- |
| BJ23-01 | 0.979 | 0.013 | 0.008 | SQ20-06 | 0.003 | 0.005 | 0.992 |
| BJ23-02 | 0.963 | 0.003 | 0.034 | SQ20-07 | 0.15 | 0.005 | 0.845 |
| BJ23-03 | 0.985 | 0.003 | 0.012 | SQ20-13 | 0.011 | 0.003 | 0.986 |
| BJ23-04 | 0.975 | 0.004 | 0.021 | SQ20-15 | 0.003 | 0.003 | 0.995 |
| BJ23-05 | 0.978 | 0.003 | 0.019 | SQ20-17 | 0.88 | 0.005 | 0.115 |
| BJ23-06 | 0.895 | 0.007 | 0.098 | SQ20-21 | 0.064 | 0.006 | 0.93 |
| BJ23-07 | 0.991 | 0.004 | 0.005 | SQ20-22 | 0.008 | 0.006 | 0.986 |
| CS20-01 | 0.929 | 0.028 | 0.044 | SQ20-23 | 0.015 | 0.006 | 0.978 |
| CS20-02 | 0.979 | 0.005 | 0.016 | TJ23-01 | 0.965 | 0.007 | 0.028 |
| CS20-04 | 0.008 | 0.003 | 0.988 | TJ23-02 | 0.99 | 0.004 | 0.007 |
| CS20-05 | 0.005 | 0.002 | 0.993 | TJ23-03 | 0.936 | 0.004 | 0.06 |
| CS20-06 | 0.031 | 0.004 | 0.965 | TJ23-04 | 0.953 | 0.035 | 0.012 |
| CS20-07 | 0.005 | 0.003 | 0.992 | TJ23-05 | 0.979 | 0.014 | 0.007 |
| CS20-23 | 0.026 | 0.005 | 0.969 | TN23-01 | 0.017 | 0.008 | 0.975 |
| DJ20-01 | 0.017 | 0.003 | 0.98 | TN23-02 | 0.01 | 0.003 | 0.988 |
| DJ20-02 | 0.006 | 0.002 | 0.992 | TN23-03 | 0.003 | 0.002 | 0.995 |
| DJ20-03 | 0.008 | 0.005 | 0.988 | TN23-04 | 0.004 | 0.003 | 0.993 |
| DJ20-04 | 0.006 | 0.002 | 0.991 | TN23-05 | 0.021 | 0.004 | 0.975 |
| DJ20-05 | 0.005 | 0.002 | 0.993 | TN23-06 | 0.004 | 0.006 | 0.99 |
| DJ20-06 | 0.006 | 0.003 | 0.992 | TN23-07 | 0.003 | 0.004 | 0.993 |
| DZ23-01 | 0.005 | 0.002 | 0.993 | TN23-08 | 0.003 | 0.003 | 0.995 |
| DZ23-02 | 0.005 | 0.002 | 0.992 | TN23-09 | 0.003 | 0.003 | 0.994 |
| DZ23-03 | 0.028 | 0.003 | 0.968 | TN23-10 | 0.03 | 0.003 | 0.967 |
| DZ23-04 | 0.094 | 0.004 | 0.902 | TN23-11 | 0.013 | 0.007 | 0.98 |
| DZ23-05 | 0.014 | 0.004 | 0.981 | TZ23-01-1 | 0.003 | 0.003 | 0.994 |
| DZ23-06 | 0.016 | 0.002 | 0.982 | TZ23-01-2 | 0.003 | 0.002 | 0.995 |
| FG22-01 | 0.992 | 0.002 | 0.006 | TZ23-01-3 | 0.004 | 0.003 | 0.993 |
| FG22-02 | 0.993 | 0.003 | 0.005 | TZ23-02 | 0.006 | 0.002 | 0.992 |
| FG22-03 | 0.99 | 0.005 | 0.005 | TZ23-03 | 0.004 | 0.002 | 0.995 |
| FG22-04 | 0.995 | 0.003 | 0.003 | TZ23-04 | 0.006 | 0.002 | 0.992 |
| FG22-05 | 0.993 | 0.003 | 0.004 | WC20-01 | 0.143 | 0.004 | 0.853 |
| FG22-06 | 0.995 | 0.002 | 0.003 | WC20-02 | 0.016 | 0.002 | 0.982 |
| FG22-07 | 0.995 | 0.002 | 0.003 | WC20-03 | 0.032 | 0.003 | 0.965 |
| FG22-08 | 0.995 | 0.002 | 0.004 | WC20-04 | 0.006 | 0.01 | 0.984 |
| GL23-01 | 0.914 | 0.007 | 0.079 | WC20-06 | 0.004 | 0.003 | 0.993 |
| GL23-02 | 0.979 | 0.011 | 0.009 | XS20-01 | 0.002 | 0.002 | 0.996 |
| GL23-03 | 0.035 | 0.021 | 0.943 | XS20-02 | 0.237 | 0.004 | 0.759 |
| GL23-04 | 0.948 | 0.012 | 0.04 | XS20-03 | 0.14 | 0.006 | 0.855 |
| GL23-05 | 0.924 | 0.006 | 0.07 | XS20-04 | 0.119 | 0.014 | 0.867 |
| GL23-06 | 0.728 | 0.003 | 0.27 | YQ22-01 | 0.979 | 0.005 | 0.015 |
| GL23-07 | 0.961 | 0.007 | 0.032 | YQ22-02 | 0.992 | 0.002 | 0.005 |
| GL23-08 | 0.912 | 0.004 | 0.085 | YQ22-03 | 0.988 | 0.002 | 0.01 |
| JH23-01 | 0.981 | 0.009 | 0.01 | YQ22-04 | 0.994 | 0.002 | 0.004 |
| JH23-02 | 0.946 | 0.004 | 0.05 | YQ22-05 | 0.984 | 0.007 | 0.009 |
| JH23-03 | 0.978 | 0.005 | 0.017 | YQ22-06 | 0.892 | 0.004 | 0.104 |
| JK20-01 | 0.976 | 0.003 | 0.021 | YQ22-07 | 0.979 | 0.004 | 0.016 |
| JK20-02 | 0.005 | 0.002 | 0.993 | YQ22-08 | 0.992 | 0.004 | 0.004 |
| JK20-03 | 0.004 | 0.003 | 0.993 | YQ22-09 | 0.984 | 0.011 | 0.005 |
| JK20-04 | 0.057 | 0.008 | 0.936 | YQ22-10 | 0.957 | 0.024 | 0.02 |
| JK20-05 | 0.037 | 0.011 | 0.951 | YQ22-11 | 0.994 | 0.003 | 0.003 |
| JK20-06 | 0.007 | 0.004 | 0.989 | ZA20-01 | 0.003 | 0.002 | 0.995 |
| JK20-07 | 0.02 | 0.003 | 0.977 | ZA20-02 | 0.03 | 0.006 | 0.964 |
| JK20-08 | 0.005 | 0.002 | 0.994 | ZA20-03 | 0.028 | 0.003 | 0.969 |
| NM23-01 | 0.012 | 0.002 | 0.985 | ZA20-04 | 0.007 | 0.006 | 0.988 |
| NM23-02 | 0.014 | 0.005 | 0.981 | ZA20-05 | 0.717 | 0.004 | 0.279 |
| NM23-03 | 0.028 | 0.004 | 0.969 | ZA20-06 | 0.022 | 0.021 | 0.957 |
| NM23-04 | 0.719 | 0.014 | 0.266 | ZG23-01 | 0.024 | 0.003 | 0.972 |
| NM23-05 | 0.007 | 0.003 | 0.99 | ZG23-02 | 0.015 | 0.002 | 0.982 |
| NM23-06 | 0.004 | 0.002 | 0.994 | ZG23-03 | 0.005 | 0.002 | 0.993 |
| NM23-07 | 0.008 | 0.002 | 0.99 | ZG23-04 | 0.018 | 0.002 | 0.98 |
| NM23-08 | 0.007 | 0.007 | 0.986 | ZG23-05 | 0.023 | 0.003 | 0.974 |
| MN23-1 | 0.037 | 0.953 | 0.009 | ZIN23-1 | 0.003 | 0.994 | 0.003 |
| MN23-10 | 0.005 | 0.991 | 0.004 | ZIN23-10 | 0.006 | 0.989 | 0.006 |
| MN23-2 | 0.009 | 0.988 | 0.003 | ZIN23-2 | 0.013 | 0.982 | 0.005 |
| MN23-3 | 0.004 | 0.992 | 0.004 | ZIN23-3 | 0.009 | 0.971 | 0.02 |
| MN23-4 | 0.262 | 0.731 | 0.007 | ZIN23-4 | 0.009 | 0.974 | 0.018 |
| MN23-5 | 0.047 | 0.937 | 0.016 | ZIN23-5 | 0.002 | 0.99 | 0.008 |
| MN23-6 | 0.005 | 0.992 | 0.003 | ZIN23-6 | 0.006 | 0.988 | 0.005 |
| MN23-7 | 0.006 | 0.985 | 0.009 | ZIN23-7 | 0.037 | 0.912 | 0.051 |
| MN23-8 | 0.018 | 0.967 | 0.016 | ZIN23-8 | 0.006 | 0.99 | 0.004 |
| MN23-9 | 0.011 | 0.983 | 0.006 | ZIN23-9 | 0.019 | 0.974 | 0.007 |
| MT20-01 | 0.015 | 0.003 | 0.982 | ZJ23-01 | 0.979 | 0.006 | 0.014 |
| MT20-02 | 0.013 | 0.003 | 0.984 | ZJ23-02 | 0.976 | 0.006 | 0.018 |
| MT20-03 | 0.405 | 0.003 | 0.592 | ZJ23-03 | 0.954 | 0.005 | 0.041 |
| MT20-04 | 0.041 | 0.004 | 0.956 | ZJ23-04 | 0.975 | 0.01 | 0.015 |
| SN10-06 | 0.168 | 0.03 | 0.802 | ZJ23-05 | 0.981 | 0.009 | 0.01 |
| SN20-02 | 0.012 | 0.003 | 0.985 | ZJ23-06 | 0.986 | 0.005 | 0.009 |
| SN20-03 | 0.012 | 0.008 | 0.98 | ZJN23-1 | 0.004 | 0.993 | 0.002 |
| SN20-08 | 0.034 | 0.008 | 0.957 | ZJN23-10 | 0.003 | 0.994 | 0.003 |
| SN20-09 | 0.979 | 0.01 | 0.012 | ZJN23-2 | 0.003 | 0.995 | 0.003 |
| SN20-11 | 0.566 | 0.005 | 0.429 | ZJN23-3 | 0.004 | 0.994 | 0.002 |
| SN20-16 | 0.006 | 0.003 | 0.991 | ZJN23-4 | 0.004 | 0.99 | 0.006 |
| SN20-17 | 0.005 | 0.002 | 0.993 | ZJN23-5 | 0.004 | 0.994 | 0.003 |
| SN20-18 | 0.077 | 0.01 | 0.914 | ZJN23-6 | 0.006 | 0.986 | 0.009 |
| SQ20-01 | 0.004 | 0.002 | 0.994 | ZJN23-7 | 0.006 | 0.991 | 0.003 |
| SQ20-03 | 0.017 | 0.005 | 0.978 | ZJN23-8 | 0.004 | 0.994 | 0.003 |
| SQ20-05 | 0.006 | 0.003 | 0.991 | ZJN23-9 | 0.004 | 0.992 | 0.004 |
