# Supplementary figures and images for: Development and characterization of SSR markers in Phoebe zhennan
Source: PeerJ. 2025 Dec 15;13:e20434. doi: 10.7717/peerj.20434 (PMC12713558; doi:10.7717/peerj.20434)

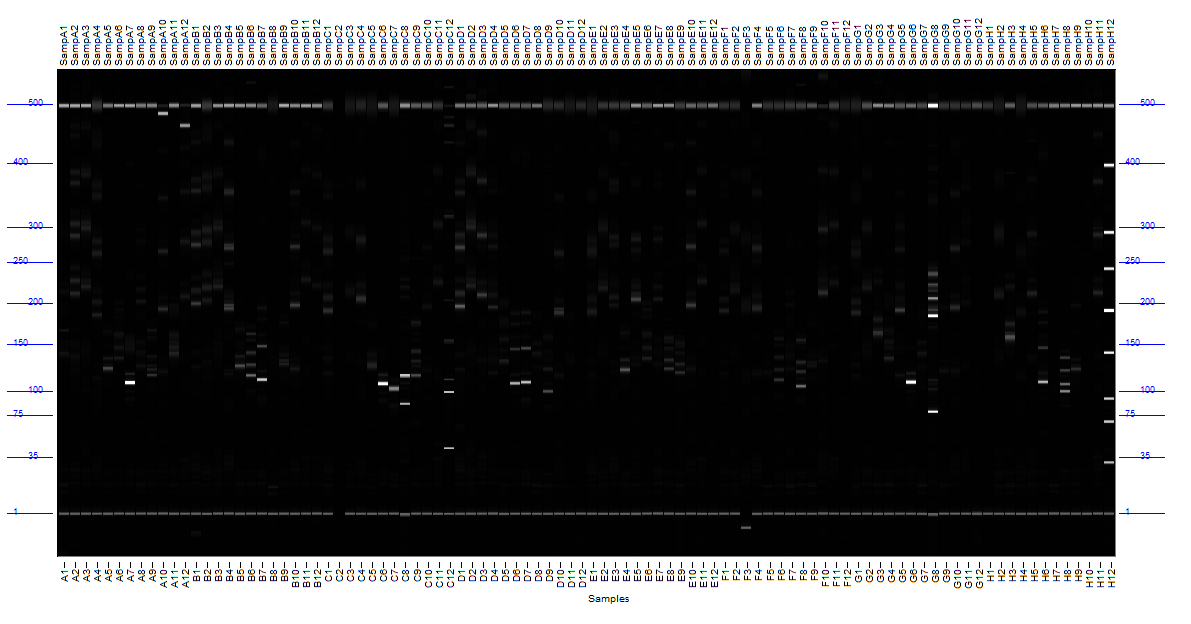

Supplement: Supplemental Information 4 [file peerj-13-20434-s004.png]
